# Impact of spatial distribution on the development of mutualism in microbes

**DOI:** 10.3389/fmicb.2014.00649

**Published:** 2014-11-24

**Authors:** Ákos T. Kovács

**Affiliations:** Terrestrial Biofilms Group, Institute of Microbiology, Friedrich Schiller University of JenaJena, Germany

**Keywords:** cooperation, non-producer, assortment, microbial population, surfaces

## Abstract

The evolution of mutualism is one of the long-standing puzzles in evolutionary biology. Why would an individual contribute to the group at the expense of its own fitness? Individual bacterial cells cooperate by secreting products that are beneficial for the community, but costly to produce. It has been shown that cooperation is critical for microbial communities, most notably in biofilms, however, the degree of cooperation strongly depends on the culturing conditions. Spatial community structure provides a solution how cooperation might develop and remain stable. This perspective paper discusses recent progresses on experiments that use microbes to understand the role of spatial distribution on the stability of intraspecific cooperation from an evolutionary point of view and also highlights the effect of mutualism on spatial segregation. Recent publications in this area will be highlighted, which suggest that while mechanisms that allow assortment help to maintain cooperative traits, strong mutualism actually promotes population intermixing. Microbes provide simple and suitable systems to examine the features that define population organization and mutualism.

Evolutionary biologists have had a long-standing interest in elucidating the mechanisms sustaining the persistence of cooperation. Why would an individual restrict its own fitness to benefit the whole population? The evolution of cooperation has extensively been studied in animals, while the advantages of research on microbes have recently been exploited to address this puzzle. Microbial cells cooperate by secreting and sharing products that benefit the community, but are costly to produce. Numerous examples have been described that exemplify cooperation in microbes and the dynamics among producing and non-producing members of the population (reviewed in West et al., 2006; Nadell et al., 2008). Importantly, the establishment of cooperation simultaneously results in strategies that ensure the stability of cooperative traits by actively or indirectly reducing the presence of cheaters (Travisano and Velicer, 2004). Microbial colonization of surfaces provides one of the solutions to maintain the stability of cooperation. As microorganisms settle and inhabit biotic or abiotic surfaces, spatial variances in nutrient composition produce subtle environmental differences that allow for a variety of ecological interactions. Cooperation is critical in certain microbial communities, most notably in biofilms, where the degree of cooperation strongly depends on the culturing conditions. In nature, most microbes persist in surface-attached sedentary communities known as biofilms. These communities provide a simple illustration of microbial complexity: distinct cells residing in a biofilm deliver cooperative traits, like the secretion of extracellular polysaccharides (EPS), and these traits are advantageous at the biofilm level, because they improve its resilience and ability to grow, while the costs arise at the single cell level within the population. Here, the focus is given to experimental systems that study intraspecific interactions.

Theoretical studies reveal the importance of microbial spatial distribution. Local environmental differences, e.g., in nutrient concentration, diversify the cell growth and alter the spatial distribution of cells within the population (Xavier and Foster, 2007). Moreover, cooperation is favored when certain parameters are present in a structured environment according to simulation (Allen et al., 2013). Small diffusion rates (low diffusion retains secreted compounds and enzymes close to producers), low colony dimensionality (flat versus complex three-dimensional structures), and small rates of decay of the commonly used and accessible metabolites (public goods) all support cooperation according to mathematical simulations (Allen et al., 2013). Local spatial differences in the microbial population composition might originate from a stochastic distribution of founder cells. Theoretical simulations suggest that EPS producers in biofilms outcompete non-producers in the presence of solute gradient (e.g., oxygen or resource gradient; Xavier and Foster, 2007). Under non-realistic conditions, when no gradient is present, the competition is purely driven by growth rate, and since non-producers are not paying the costs, their growth rate is superior. The competitive advantage of EPS producers originates from the benefit of EPS used only locally. Simulations suggest that when cells in a biofilm population start to grow, EPS production results in lower growth rates (Xavier and Foster, 2007). In later stages, EPS secretion actually helps the producer cells to push the progeny out from the focal cell layer. This process presents a primitive form of kin selection, where cells provide benefits to their descendants, as EPS is most likely shared among cells that are close to each other. While at the population level a variety of microbes are present with low relatedness, locally, highly related clonal population might exist, affecting mathematical simulations, and evolutionary outcome (Xavier and Foster, 2007). Secretion of EPS by a cell allows it to altruistically push its descendants into a more nutrient-rich environment. There is a fascinating analogy to plants here where vertical growth and leaf coverage increase access to light at the expense of competitors (Xavier and Foster, 2007). Simulations suggest that negative frequency-dependent selection, i.e., the fitness of a phenotype increases as it becomes rarer, is also relevant in microbial populations, where both EPS producers and non-producers are able to invade the population primarily made of cells following the other strategy; however, cooperation will be stabilized long-term at the population level (Xavier and Foster, 2007). Thus, EPS production is an altruistic behavior, as secreting cells have lower growth rates and division, but aid other cells (mostly their descendants) to reach nutrient rich sectors. EPS production in these cases provides a selective advantage by allowing the producer population to rise up and over other cells, therefore reaching more nutrients or oxygen and suffocating others that are left behind.

The consequence of EPS production on cooperation has been further studied in various experimental approaches. In *Vibrio cholerae* chitinase production is exploited by non-producers in mixed environments, while production of thick biofilms or increased flow rate could solve the public good problem, i.e., how cooperative traits are stably maintained in microbes (Drescher et al., 2014). Specifically, thick biofilm allows cooperators to sequester all liberated sugars (public goods) to themselves, while flow removes the majority of liberated sugars so the exploitative non-producer cells cannot use them. Various initial ratios of producers versus non-producers were examined and it was suggested that under mixed conditions (shaked culture) a higher ratio of producers helps the population as a whole resulting in higher growth rate, while the non-producers grow more rapidly by not paying the metabolic cost of chitinase production. Growing on a surface itself does not solve the public good dilemma, as deliberated sugars distribute within the chamber, even at low initial founder cell densities. A solution is provided when cells form thick biofilms. In such a situation, cells far away from the chitin source or outside of the biofilm experience low concentrations of liberated sugars while those cells that inhabit the thick biofilm of producers will benefit from the available resources. Further, biofilm matrix production also increases spatial segregation. Flow of the surrounding medium clarifies the public good dilemma in a different way: in the presence of a current in the medium, all cells experience a reduced concentration of public goods (Drescher et al., 2014). This is selectively disadvantageous for the non chitinase producers because these cells do not benefit from chitin degraded by chitinase in their intimate vicinity, while producers still harvest enough resources close to the enzyme production site.

Excretion of EPS, although benefitting producers, comes with a fitness trade-off. The experiments of Nadell and Bassler (2011) using *V. cholerae* biofilms showed that while EPS production locally benefits clonal cells and gain dramatic advantage, it has an ecological cost in the form of restricted dispersal. EPS producers are impaired in their ability to escape from the biofilm and colonize new niches, presenting a trade-off on matrix production. Their experiments comprised a flagellum and quorum-sensing (QS) deficient strain that constitutively produces EPS (EPS+) and an isogenic but *vpsL* variant that is not producing the biofilm matrix any more (EPS-). While QS mutant *hapR* produces EPS in a constitutive manner, the *vpsL* mutant is highly motile. To examine the sole effect of EPS producing ability, a flagellin A (*flaA*) mutation was introduced into both strains and they were labeled with a fluorescent dye that had no fitness effect on the growth. While in liquid culture, the EPS- cells have a benefit when co-cultured with the EPS+ strain, however, under biofilm conditions, EPS+ cells increase more rapidly in number depending on their initial fraction. One of the advantages could be that the production of the matrix helps the cells to stick to the substratum and resist shared stress (Nadell and Bassler, 2011). EPS+ cells benefit themselves and their daughter cells similarly to what was suggested by the simulation described above. Therefore each tower like biofilm structure predominantly contains cells of one linage. This is also true if genetically identical EPS+ strains are used to initiate flow cell biofilms of *V. cholera*, labeled with different fluorescent reporter proteins. This might explain why EPS production could develop in nature and how exploitation by non-producers might be avoided. However, local competition is not the only factor contributing to long term evolutionary dynamics. The liquid eﬄuent was also monitored during the experiments. While EPS producers were major part of the biofilm obtained in a microfluidic device, the eﬄuent mainly contained EPS- cells at weak but also under strong disturbance. Therefore the competition between EPS+ and EPS- cells also depends on how often empty spots are available that are colonized by dispersing clones (Nadell and Bassler, 2011). Therefore the regulation of both biofilm formation and dispersal is important from an evolutionary point of view. *Staphylococcus aureus* is an important example where after establishing dense community, biofilm gene expression is decreased in a QS [i.e., accessory gene regulator (Agr) system] dependent manner and dispersal is activated (Boles and Horswill, 2008).

Spatial population expansion was recently suggested to also facilitate the evolution of cooperation (**Figure 1**). The studies of van Dyken et al. (2013) and van Gestel et al. (2014) used colonies of *Saccharomyces cerevisiae* and *Bacillus subtilis*, respectively, to demonstrate the impact of assortment within the population on public good exploitation by non-producers. The experiments on *Saccharomyces cerevisiae* employed a simple system where microbial genetic drift can be followed. In this colony expansion system, described first by Hallatschek et al. (2007), radial growth from a founder area depends on cell division mediated expansion (i.e., daughter cells push neighboring cells). As cells deplete the nutrients at the colony’s edge, only few cells contribute to propagation leading to a series of genetic bottlenecks that causes high local fixation probability of clonal linages. Theory predicts that cooperation is favored at high genetic relatedness of the microbial cells (van Dyken et al., 2013). However, non-producers might arise rapidly within a population stochastically fixed at the front and increase in number during expansion. The sucrose invertase secretion ability of *Saccharomyces cerevisiae* was employed to examine the dynamics between fluorescently marked strains of public good producers (cooperators) and non-producers in expanding colonies (van Dyken et al., 2013). The exoenzyme sucrose invertase catalyzes the digestion of sucrose to monosaccharides, which can be imported by the cells and metabolized. Under well-mixed unstructured conditions, cooperators decline at all initial frequencies due to the cost of cooperative trait production, in spite of the growth advantage in pure cultures comprising cooperators only. In contrast, during radial colony growth on the surface (i.e., structured environment), cooperators increase in frequency as expansion proceeds. Cooperators also invade non-cooperator populations when initiated at low frequency, and resist public good exploitation by non-producers in later stages due to a faster spreading ability of the producer strains. Thus, under these conditions, high assortment reduces the direct local competition between cooperators and non-producers, and therefore diminishes the potential benefit of defection (van Dyken et al., 2013). As cooperators establish themselves locally, their productivity is higher resulting in a fitness advantage outweighing the costs of cooperation. This leads to a further increase in cooperator frequency.

**FIGURE 1:**
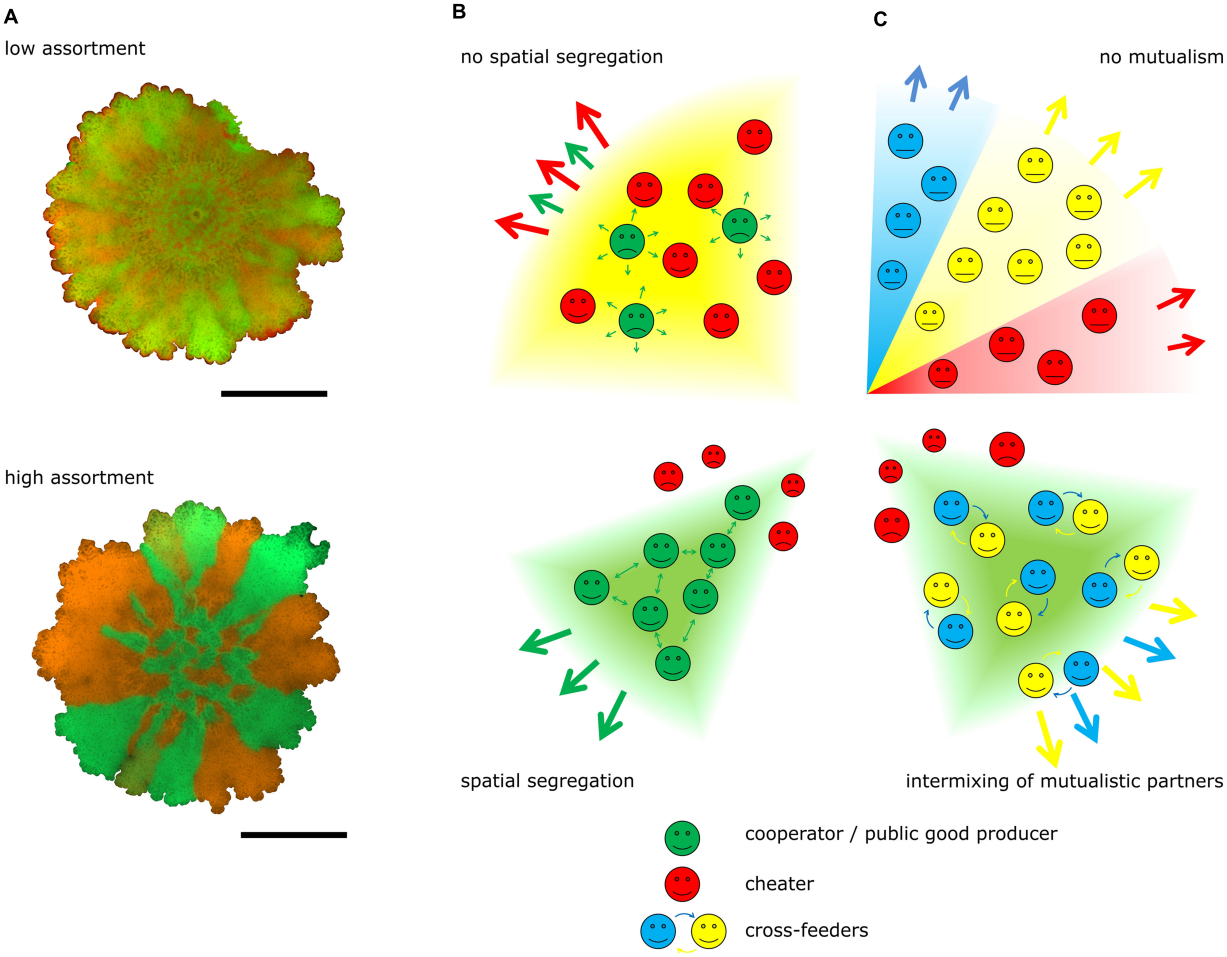
**Cooperation and spatial distribution. (A)** Experimental examples of *Bacillus subtilis* colony biofilms are shown with low (above) and high (below) spatial assortment of genetically identical strains with different fluorescent labels. Strains producing green- or red-fluorescence proteins were mixed and used to initiate colony biofilms as described by van Gestel et al. (2014). Pictures were obtained with AxioZoom V16 microscope (Zeiss). Scale bars indicate 5 mm. **(B)** Schematic figures depict the role of spatial segregation on the stability of cooperation: non-producers exploit the public good produced by cooperators if no spatial segregation exists in the expanding population (above), while cooperative traits are sustained under high spatial segregation (below). **(C)** Spatial segregation during competition (above) is suppressed when microbial strains or species are exchanged in a mutualistic interaction (below), which results intermixing of partners. Green and red symbols indicate public good producers and non-producers, respectively, while blue and yellow symbols denote mutualistic cross-feeders. Thick arrows show colony expansion, whereas thin arrows designate public **(B)** or exchanged **(C)** goods.

Spatial pattern formation in *B. subtilis* biofilms was examined using a distinct system (van Gestel et al., 2014). Colony expansion of *B. subtilis* depends on the production of EPS, a biofilm component with fitness costs associated with the production. Genetic drift observed for *Saccharomyces cerevisiae* was not observed in colony biofilms of *B. subtilis*. However, spatial patterns can be modulated on a continuous scale by altering the founder cell density. Mathematical modeling and experimental approaches both showed that higher dilution of initiating cell numbers increases the degree of assortment that occurs during colony biofilm development, while low dilution, i.e., high founder cell density, results in reduced spatial segregation (van Gestel et al., 2014). Competition experiments that exploit the possibility to alter the level of assortment were employed to show that EPS-producing cells have a selective advantage over non-cooperative mutants when colonization occurs at high spatial segregation, while they have a disadvantage when assortment is low. In addition, adjusting EPS production to diverse levels indicated that the level of EPS production in the wild type cells facilitates surface colonization at an optimal level and secretion of surplus matrix does not aid to further the expansion properties (van Gestel et al., 2014). These experiments therefore showed that colonization dynamics of complex microbial communities could determine the persistence of cooperation.

As discussed above, structured environments define spatial distribution of microbes, which, in turn, impacts the stability of cooperation. From a different perspective, ecological interactions might also define pattern formation and, therefore, the degree of mixing (**Figure 1**). Several recent studies showed that strong mutualistic interactions can stimulate partner intermixing where spatial segregation is suppressed (Momeni et al., 2013a,b; Müller et al., 2014). Using the yeast colony expansion system described above, Müller et al. (2014) genetically engineered two cross-feeding yeast strains and followed the degree of genetic drift in expanding colonies. In these experiments, growth of auxotroph mutants depends on the metabolites excreted by a producer strain, and *vice versa*. They found that nutrient rich medium allows spatial segregation (i.e., distinctive sectors are observed corresponding to the different genotypes). However, when a nutrient poor medium was employed where cell growth and therefore colony expansion depends on cross-feeding, intertwinned patches of the strains could be observed during spatial expansion as mutualism requires physical proximity of the interacting partners. The degree of intertwinement was suggested to be related to the diffusion properties of the mutualism compounds. Genetic drift and mutualism are opposing forces, as one separates, while the other mixes the members of the microbial community, respectively (Müller et al., 2014). Mutualism is the driving force only until a certain nutrient concentration is reached. From then on, genetic drift has the higher impact on colony expansion and community structure. By varying the concentration of different cross-feeding metabolites, the degree of mixing could be examined. At concentrations where mutualism would be still beneficial (i.e., level of nutrient that still enhances growth of the auxotroph strain in pure culture), genetic drift already outcompetes mutualism (i.e., in these experiments, 25% of the minimal nutrient concentration that supports maximal growth rate). Thus mutualistic microorganisms can expand only if the benefit of mutualism is sufficiently strong and if dispersal of the partners is sustained (Müller et al., 2014). It is plausible to assume that the degree of assortment and mutualism driven intermixing also fluctuates depending on the local environmental conditions.

Mutualism driven partner intermixing is also maintained in surface-grown communities where microbes settle at distinct spatially distributed spots and expand to colonize the available niche. Momeni et al. (2013a) utilized metabolite-exchanging yeast strains with different degrees of interactions (i.e., from commensalism to cooperation), and showed that robust mutualism leads to partner intermixing, when ecological interactions are the major patterning factor. Such a stable mutualistic community can return to a stable population composition after it is disturbed. Conversely, competing populations with no metabolic interdependence tend to segregate, described as competitive exclusion (Momeni et al., 2013a). Simulations and analytical calculations based on three-dimensional fitness models with different interaction scenarios showed that initial partner ratios can converge over time if the interaction benefits at least one of the partners, but not for competitive communities. Also, these simulations predict partner intermixing for strong cooperation. Interestingly, further experiments revealed that mixing of mutualistic populations results in a layered pattern where the intermixing index increased proportionally as a function of community height. As observed for colony expansion, layering of the yeast communities, and local patch sizes were suggested to be determined by the localized nutrient supply and consumption, i.e., the distance a secreted nutrient can diffuse within the community. Curiously, while initial partner ratio did not significantly affect the level of intermixing, at very high initial cell densities, intermixing was also observed in the absence of cooperation (Momeni et al., 2013a).

Community patterning is also influenced via partner fidelity feedback, where non-producers are unable to evade cooperator populations. Again, a combination of experiments on yeast and mathematical modeling was applied by Momeni et al. (2013b) to inspect partner fidelity feedback in surface-attached structured environments. Two cross-feeding strains were mixed with a non-producer strain that consumes one of the metabolites produced by the mutualistic cooperators, but not releasing any public good. Non-producers had increased fitness in an unstructured, well-mixed environment. On the contrary, in a structured environment, the cooperators self-organized into mixed clusters, as above, while the cheating strain was excluded from these clusters of mutualistic cells. Partner choice could be ignored in this phenomenon as yeast cells are incapable of partner recognition (Momeni et al., 2013a). Additionally, simulations suggested that self-organization of the mutualistic partners and exclusion of non-producers are driven by asymmetric fitness effects, i.e., unequal spatially localized benefits that the partners supply to the heterotrophic partner during cell growth and expansion. Isolation of non-producers therefore enabled cooperators to rise in frequency regardless of the intrinsic advantage of non-producers over cooperators (Momeni et al., 2013a).

The experiments above all point to the eligibility of microbial systems to demonstrate basic evolutionary theories. These experiments clearly support the idea that genetic drift maintains cooperation within clonal linages; intermixing supports close mutualism in structured environment and in some cases, intermixing with preferred partners might even promote exclusion of non-producers. All these experiments demonstrate that spatial self-organization provides a solution for stability of intraspecific cooperation without the need for specific molecular mechanism for partner recognition. Experimental microbial systems greatly help us to understand and emphasize the importance of ecology and significance of spatial structures for the evolution of cooperation.

## Conflict of Interest Statement

The author declares that the research was conducted in the absence of any commercial or financial relationships that could be construed as a potential conflict of interest.
